# *Tgfbr2* in Dental Pulp Cells Guides Neurite Outgrowth in Developing Teeth

**DOI:** 10.3389/fcell.2022.834815

**Published:** 2022-02-21

**Authors:** Monica Stanwick, Courtney Barkley, Rosa Serra, Andrew Kruggel, Amy Webb, Yue Zhao, Maciej Pietrzak, Chandler Ashman, Allie Staats, Shifa Shahid, Sarah B. Peters

**Affiliations:** ^1^ Division of Biosciences, College of Dentistry, The Ohio State University, Columbus, OH, United States; ^2^ Department of Cell Developmental and Integrative Biology, University of Alabama at Birmingham, Birmingham, AL, United States; ^3^ Department of Biomedical Informatics, The Ohio State University, Columbus, OH, United States

**Keywords:** TGFβ, semaphorins, dental pulp, tooth innervation, neurite outgrowth

## Abstract

Transforming growth factor β (TGFβ) plays an important role in tooth morphogenesis and mineralization. During postnatal development, the dental pulp (DP) mesenchyme secretes neurotrophic factors that guide trigeminal nerve fibers into and throughout the DP. This process is tightly linked with dentin formation and mineralization. Our laboratory established a mouse model in which *Tgfbr2* was conditionally deleted in DP mesenchyme using an Osterix promoter-driven Cre recombinase (*Tgfbr2* ^*cko*^). These mice survived postnatally with significant defects in bones and teeth, including reduced mineralization and short roots. Hematoxylin and eosin staining revealed reduced axon-like structures in the mutant mice. Reporter imaging demonstrated that Osterix-Cre activity within the tooth was active in the DP and derivatives, but not in neuronal afferents. Immunofluorescence staining for β3 tubulin (neuronal marker) was performed on serial cryosections from control and mutant molars on postnatal days 7 and 24 (P7, P24). Confocal imaging and pixel quantification demonstrated reduced innervation in *Tgfbr2* ^*cko*^ first molars at both stages compared to controls, indicating that signals necessary to promote neurite outgrowth were disrupted by *Tgfbr2* deletion. We performed mRNA-Sequence (RNA-Seq) and gene onotology analyses using RNA from the DP of P7 control and mutant mice to investigate the pathways involved in *Tgfbr2*-mediated tooth development. These analyses identified downregulation of several mineralization-related and neuronal genes in the *Tgfbr2* ^*cko*^ DP compared to controls. Select gene expression patterns were confirmed by quantitative real-time PCR and immunofluorescence imaging. Lastly, trigeminal neurons were co-cultured atop Transwell filters overlying primary *Tgfbr2* ^*f/f*^ DP cells. *Tgfbr2* in the DP was deleted *via* Adenovirus-expressed Cre recombinase. Confocal imaging of axons through the filter pores showed increased axonal sprouting from neurons cultured with *Tgfbr2*-positive DP cells compared to neurons cultured alone. Axon sprouting was reduced when *Tgfbr2* was knocked down in the DP cells. Immunofluorescence of dentin sialophosphoprotein in co-cultured DP cells confirmed reduced mineralization potential in cells with *Tgfbr2* deletion. Both our proteomics and RNA-Seq analyses indicate that axonal guidance cues, particularly semaphorin signaling, were disrupted by *Tgfbr2* deletion. Thus, *Tgfbr2* in the DP mesenchyme appears to regulate differentiation and the cells’ ability to guide neurite outgrowth during tooth mineralization and innervation.

## Introduction

The transforming growth factor β (TGFβ) superfamily regulates many developmental processes, including those that occur in the tooth and skeleton (Oka et al., 2007; Seo and Serra, 2009; Sohn et al., 2010; Wang et al., 2013; Zhen et al., 2013; Iwata et al., 2014; Choi et al., 2016; Peters et al., 2017; Niwa et al., 2018; Kawatsu et al., 2021; Zhang et al., 2021). Tooth development requires the coordination of epithelial, mesenchymal, hematopoietic, and neuronal cell populations to construct a functional sensory organ. Tooth development begins with an invagination of the ectodermal epithelium into the surrounding mesenchyme, around embryonic day 11 in the mouse. The epithelial bud then bifurcates and elongates. The underlying dental mesenchyme later gives rise to the dental pulp (DP) and dentin-producing odontoblasts. During postnatal development, afferent axons from the trigeminal ganglia (TG) penetrate into and throughout the tooth around the time dentin deposition begins [reviewed in (Pagella et al., 2014); (Shadad et al., 2019)]. Innervation rapidly progresses to arborize into sub-odontoblastic, odontoblastic, and predentinal axonal networks, with axons present in coronal dentin canals (Byers, 1980). This sensory innervation provides signals crucial to daily oral activities, such as eating and talking, and provides pain signals to protect the tooth organ (Hildebrand et al., 1995). The roles of TGFβ signaling in early aspects of tooth development and pathology were previously documented in mice with mutations or targeted deletions of genes encoding components of TGFβ signaling pathways (Ko et al., 2007; Zhao et al., 2008; Cho et al., 2013; Wang et al., 2013; Peters et al., 2017). However, *Tgfbr2* deletion in the dental and skeletal mesenchyme or epithelium is embryonic or perinatal lethal (Dünker and Krieglstein, 2002; Ito et al., 2003; Baffi et al., 2004; Seo and Serra, 2007, 2009; Zhao et al., 2011; Iwata et al., 2012). Since axons from the trigeminal ganglion do not penetrate the DP until around P3 (Moe et al., 2012; Shadad et al., 2019), this lethality prevented comprehensive research into the roles of TGFβ signaling in tooth innervation.

To address this obstacle, our laboratory established an Osterix promoter-driven Cre recombinase mouse model in which *Tgfbr2* was conditionally deleted in the osteoblast- and odontoblast-producing mesenchyme (*Tgfbr2* ^*cko*^) (Wang et al., 2013; Peters et al., 2017; Corps et al., 2021). The *Tgfbr2* ^*cko*^ mice did not demonstrate skeletal or dental abnormalities at birth, but within the first week of life began to demonstrate reduced growth and mineralization in bones and teeth that worsened with development. The mutant phenotype included stunted root elongation and hypomineralization in molars (Wang et al., 2013; Peters et al., 2017; Corps et al., 2021). We stained P5, P7, P10, and P14 mandibular first molars (M1s) with hematoxylin and eosin (H&E) to investigate the short tooth root phenotype and noticed a reduction in axon-like structures in the mutant mice. Since Osterix-Cre should be restricted to the mesenchymal lineage (Rodda and McMahon, 2006; Rakian et al., 2013; Wang et al., 2013; Ono et al., 2016; Peters et al., 2017), neuronal alterations were unexpected. However, previous studies indicated that DP cells (DPCs) secrete neurotrophic factors to regulate tooth innervation (Kettunen, 2005; Kettunen et al., 2007; Moe et al., 2012; Sijaona et al., 2012; de Almeida et al., 2014) although little information was available regarding the signaling pathways that guide this phenomenon. Furthermore, many of the previous studies focused on embryonic stages when the DP secretes repellants to prevent neurite extensions from entering the tooth rather than postnatal stages when the DP secretes axonal attractants (Lillesaar and Fried, 2004; Fried et al., 2007). We hypothesized that *Tgfbr2* in the DP mesenchyme regulates paracrine signals to guide postnatal tooth innervation.

We herein demonstrate by quantitation of a neuronal marker, β3 tubulin (β3T), that innervation was reduced in molars from *Tgfbr2* ^*cko*^ mice compared to controls starting at P7 and continuing to P24. We confirmed that the Cre expression was isolated to the dental pulp mesenchyme by crossing Osterix-Cre with the ROSA26mTmG reporter line (Rakian et al., 2013; Wang et al., 2013; Ono et al., 2016). To investigate the *Tgfbr2-*related pathways involved in tooth development, we performed an RNA-Seq analysis on DP tissue, including odontoblasts, from control and *Tgfbr2* ^*cko*^ P7 mice. Gene ontology analyses identified several overrepresented neuronal genes in the mutant mice compared to the controls, and these findings correlated with the phenotype seen in the H&E and β3T images. Subsequent qPCR analyses of RNA extracted from P7 first molars confirmed the findings for selected genes from the RNA-Seq. We then performed a co-culture using dispersed cells from the TG and primary DPCs and showed that neurite outgrowth was reduced when *Tgfbr2* was deleted. Lastly, a global evaluation of our RNA-Seq, proteomics on cell culture media, and immunofluorescence images demonstrated the existence of a developmental program regulating tooth mineralization and innervation that involves semaphorin signaling (Lillesaar and Fried, 2004; Sijaona et al., 2012). These data reveal a novel function for *Tgfbr2* in the development of mineralized, innervated teeth.

## Materials and Methods

### Mice

All experiments with mice were approved by the UAB and OSU Institutional Animal Care and Use Committees. The Osterix-Cre and *Tgfbr2* ^*cko*^ mice were described previously (Wang et al., 2013; Peters et al., 2017). Trigeminal neuron bundles for co-cultures were collected from 6-week-old B6.Cg-Tg (*Thy1*-*YFP*)16Jrs/J male and female mice. The Cre reporter strain Gt (ROSA)26SOR^tm4(ACTB-tdTomato,-EGFP)^ Luo/J (ROSA26mTmG) was crossed with Osterix-Cre mice to generate Osterix-Cre; ROSA26-mTmG mice with traceable Cre activity (Muzumdar et al., 2007). Equivalent numbers of males and females were used in all experiments.

### Imaging of DP Structures

Postnatal mandibles were fixed in 4% paraformaldehyde for 1 h, followed by EDTA decalcification. Tissue was embedded and frozen in optimal cutting temperature compound and sectioned on the sagittal plane with a cryostat (Leica Biosystems, Buffalo Grove, IL, United States) at 10 μm thickness. For immunofluorescence studies of β3T and CD31, sections were permeabilized with cold acetone at −20°C for 10 min and rinsed twice with phosphate-buffered saline (PBS). The sections were immersed in hot citrate buffer for antigen retrieval and rinsed in PBS. Histology slides were similarly immersed in hot citrate buffer and then stained with hematoxylin and eosin. Permeabilization for myopalladin and Sema3a was performed with 0.5% Tween added to the blocking solution. For all immunofluorescence studies, slides were blocked with 10% bovine serum albumin (BSA), 5% goat serum and Mouse on Mouse blocking serum (Vector Laboratories, MKB-2213-1) in PBS-Tween for 1 h at room temperature, followed by incubation overnight at 4°C in antibody dilutions: mouse anti-β3T (1:100, R&D Systems MAB1195), rabbit anti-Sema3a (1:2000, Abcam ab199475), rabbit anti-Myopalladin (1:100, Bioss Bs-10366), or rat anti-CD31 Alexafluor 647 direct conjugate (1:250, Biolegend 102516). Sections were rinsed and incubated with goat anti-mouse, goat anti-rabbit, IgG (1:500, Invitrogen) or DAPI (1:1000, Thermo Scientific) overnight at 4°C. Ten-micron thick z-stacks were obtained by confocal microscopy and presented in maximum projection mode, quantified for total pixels and normalized to the DAPI pixels using FIJI software (Schindelin et al., 2012). Unpaired t-tests with significance levels set to *p* < 0.05 were used to evaluate the significance of differences in β3T levels among four biological replicates of each genotype for P7 and P24 first molars. These tests were also used to assess the differences in CD31 among three biological replicates at multiple regions in P7 first molars.

The Comparative Pathology and Digital Imaging Shared Resource of The Ohio State University Comprehensive Cancer Center performed immunohistochemistry (IHC) on heads from P24-P27 WT and *Tgfbr2* ^*cko*^ mice. Whole heads were fixed for a minimum of 48 h in 10% neutral buffered formalin. Following decalcification using Formical-2000 (StatLab, McKinney, TX, United States), the heads were embedded in paraffin and sectioned horizontally at 4 μm thickness. Slides were dried overnight, then deparaffinized and immersed in hot citrate buffer (Dako, S1699) for antigen retrieval. A Lab Vision 360 Autostainer (Thermo Scientific, Waltham, MA, United States) was used for IHC to apply rabbit-anti-CD31 (1:900, Abcam ab281583) diluted in Dako antibody diluent (S3022) followed by donkey-anti-rabbit IgG (1:500, Jackson Immunoresearch 711-065-152), VECTASTAIN Elite ABC-HRP Reagent Peroxidase (Vector Laboratories, PK-7100), and DAB (Dako, K3468) using a standard protocol. Nuclei were stained with hematoxylin and slides were mounted for imaging. A Lionheart LX microscope (BioTek, Winooski, VT, United States) was used for imaging at ×10 magnification. IHC Images were deconvoluted and thresholded according to a previous report (Crowe and Yue, 2019). Thresholded images were quantified for total pixels, normalized to DAPI using FIJI software and analyzed with an unpaired *t*-test. Significance was set to *p* < 0.05 for 5 WT and 6 *Tgfbr2* ^*cko*^ biological replicates.

### RNA Isolation

Dental pulp was extracted from the mineralized tissue and enamel organ of M1s from P7 Osterix-Cre and *Tgfbr2* ^cko^ mice. The left and right M1s were compiled in lysis buffer (Qiagen, 79216) and dispersed with a motorized, sterile pestle, then RNA was extracted with a column isolation kit (Zymo Research, R2070) and analyzed in subsequent qPCR and RNA-Seq experiments.

### mRNA-Sequence Analysis

The RNA Integrity Number (RIN) and quantification for total RNA were determined using a BioAnalyzer RNA 6000 Pico Kit (Agilent, 5067-1513) and Qubit RNA HS Assay kit (Invitrogen, Q32852), respectively. Samples with a RIN > 7 were used for global preamplification for the 1-ng low-input RNA-seq using the SMAR-seq HT kit (Clonetech, 634437). The resultant cDNA samples were used for library generation using the Nextera XT DNA Library Prep Kit (Illumina, 15031942) and sequenced on an Illumina NovaSeq SP flowcell for 12–15 million PF clusters per sample. This protocol was described in (Lucas et al., 2019; Walker et al., 2020). Data processing and analysis were performed using a previously published pipeline (Gadepalli et al., 2019). Briefly, raw reads were aligned to a mouse reference genome (GRCh38) using HISAT2 (Liao et al., 2014; Kim et al., 2015). Gene expression values were quantified using featureCounts with GENCODE M14 (mouse) as the transcript reference (GENCODE annotation). The data were processed using the CLEAR workflow, which identifies reliably quantifiable transcripts in low-input RNA-seq experiments (Walker et al., 2020). Processed counts were transformed and normalized by voom, and a differential gene expression analysis was performed using R package “limma” (Ritchie et al., 2015). The original contributions presented in the study are publicly available. This data can be found here: NCBI, GSE190582. A gene ontology analysis was subsequently performed using Panther GO biological process complete statistical overrepresentation testing (www.pantherdb.org), employing Fisher’s exact test with false discovery rates for genes with ≥ ± 2-fold difference and *p* < 0.05.

### Quantitative Real-Time PCR

The qPCR analysis was performed according to the TaqMan^®^ PreAmp Master Mix Kit Protocol (4384557B). In brief, 20 ng of total RNA was retrotranscribed using a High Capacity cDNA Reverse Transcription kit (Life Technologies, 4374967). The samples were enriched for a pool of selected targets of interest (including the housekeeping genes), reported in Supplementary Table S1, by mixing 2.5 μl of cDNA with a pool of the Gene Expression TaqMan assays, which were mixed and diluted to ×0.2 using ×1 TE Buffer and the TaqMan PreAmp Master Mix (×2, 4391128). A total of 14 preamplification cycles were applied, then the product was diluted 1:20 with ×1 TE Buffer. A 2 μl aliquot of amplified cDNA was used to perform the qPCR experiment, with samples run in triplicate, including no-template controls. All samples were run and analyzed using the QuantStudio 12K Flex Real-Time PCR System (ThermoFisher). The Ct Average of each triplicate sample was used to perform the relative quantification analysis. The samples were normalized using the housekeeping gene, beta 2 microglobulin (B2MG) and the relative expression was calculated using the comparative Ct method. qPCR was performed on 10 Osterix-Cre M1 pairs and 10 *Tgfbr2* ^*cko*^ M1 pairs. Primer information is available in Supplementary Table S1.

### Co-Culture of TGNs and Primary DPCs

Co-culture assays were performed as previously described in (Barkley et al., 2020). Briefly, M1s from P5-8 *Tgfbr2* ^fl/fl^ mice were dispersed, plated and grown to confluence in alpha-minimum essential medium (Gibco, 12571063) supplemented with 10% heat-inactivated fetal bovine serum, L-glutamine (Gibco, 25030-081), and antibiotics. At confluence, the media were replaced with media containing Adenovirus-Cre-GFP (Ad-Cre) or Adenovirus-eGFP (Ad-GFP) in the presence of 10 mg/ml Polybrene (Millipore, TR-1003-G). After 48 h of infection, the media containing virus were removed and replaced with fresh media. NuncBlue (Invitrogen, 2272577) was added to label nuclei per the manufacturer’s instructions. Infection was confirmed by GFP fluorescence. The infection percentages were determined using NIS-Elements Advanced Research software in which an overlay of DAPI and GFP images was used to calculate the numbers of total and infected cells.

Cells from trigeminal nerve bundles were collected from 6-week-old B6. Cg-Tg (*Thy1*-*YFP*) 16Jrs/J male and female mice and dispersed with type II collagenase (Fisher, 17101015) followed by type II trypsin (Sigma, T-7409). Cells were counted using a standard hemocytometer. Transwell inserts (Greiner Thincerts, 3 μm porosity, 19380216) were coated with 10 μg/ml laminin (Sigma, L2020) and seeded with 50,000 cells/250 μl. Media were replaced with media containing 1 mM uridine (Sigma, BCCC 2099) and 15 mM 5′-fluor-2′deoxyuridine (Sigma, SLCB5571) for mitotic inhibition of mesenchymal cells within the mixture after 24 h. Co-culture continued for an additional 4 days.

*Tgfbr2* knockdown in the co-culture model was confirmed using our previously-developed semi-qPCR assay (Seo and Serra, 2007; Peters et al., 2017) (forward 5′-TTA ACA GTG ATG TCA TGG CCA GCG-3′ and reverse 5′- AGA CTT CAT GCG GCT TCT CAC AGA-3′) and B2MG (forward 5′-GCT ATC CAG AAA ACC CCT CAA-3′ and reverse 5′- CAT GTC TCG ATC CCA GTA GAC-3′), which was performed for four separate experiments.

### Primary DP Cell Staining, Imaging and Quantification

To determine the morphological responses to *Tgfbr2* knockdown in the primary DPCs with Ad-Cre infection, cells were grown atop Poly-D-lysine-coated coverslips during the co-culture assay. For immunofluorescence, cells were fixed with 4% PFA followed by permeabilization with 0.5% Tween added to a blocking solution of 10% BSA, 5% goat serum and Mouse-on-Mouse blocking serum in PBS. Primary incubation with mouse anti-DSPP (1:100, Santa Cruz, sc-73632) proceeded overnight at 4°C. The following day, the coverslips were rinsed and incubated with goat anti-mouse secondary antibody (1:500, Invitrogen) and DAPI (1:1000, Thermo Scientific) overnight at 4°C. Fluorescent images were captured and quantified with FIJI software. DSPP was normalized to the cell number. A two-tailed, unpaired *t*-test was applied to four separate experiments with values *p* < 0.05 considered to be significant.

### Assessment of Neurite Outgrowth: Staining, Imaging and Quantification

Transwell filters containing TG outgrowths were rinsed with ×1 PBS, fixed with 4% PFA, and blocked for 1 h at room temperature. Filters were then incubated at 4°C overnight with a 1:200 dilution of chicken anti-GFP primary antibody (1:200, Aves Lab, GFP-1010) to selectively stain the YFP+ neurons (due to the high conservation between GFP and YFP). Filters were then rinsed and incubated in anti-chicken IgY-H-L secondary antibody conjugated to Alexa-546 (1:500, Invitrogen, A32931). The high YFP levels in the late postnatal TG bundles resulted in high specificity for the antibody above the background fluorescence from mesenchymal cells and filter pores.

Subsequently, 100 μm-thick z-stacks were collected and stitched together in NIS-Elements. The resultant image was presented in maximum projection mode to demonstrate total neurite outgrowth throughout the entire Transwell filter. Images were auto-thresholded with the Intermodes auto-threshold function in the FIJI software, and the total numbers of pixels were quantified. A 2-way ANOVA was applied to four separate experiments with significance set to *p* < 0.05, and then a Tukey’s multiple comparison post-hoc test was performed.

### Proteomics Analysis on Co-Culture Media

The global proteomic analysis was carried out as previously reported in (Ludwig et al., 2016). In brief, cell culture media was collected at the end of the 5 days assay. A 100 μl aliquot was passed through a bone serum albumin depletion column (Genway, GWB-BSAIGY) and 40 μg of post-depleted protein was subsequently used for each GelC run. Protein samples were reduced with DTT and denatured at 70°C for 10 min prior to loading onto 10% Bis-Tris Protein gels and being fully separated. The gels were stained overnight with colloidal Coomassie for visualization purposes, then each lane was cut into 4-MW fractions and equilibrated in 100 mM ammonium bicarbonate. Each gel plug was digested overnight with mass spectrometry-grade Trypsin Gold (Promega, V5280) following the manufacturer’s instructions. Peptide extracts were reconstituted in 0.1% Formic Acid/ddH_2_O at 0.1 μg/μl. Mass spectrometry was carried out, and the data were processed, searched, filtered, grouped, and quantified as previously reported (Ludwig et al., 2016). Unpaired *t*-tests were performed comparing the co-cultures, with significance set to *p* < 0.05. The mass spectrometry proteomics data have been deposited to the ProteomeXchange Consortium *via* the PRIDE [1] partner repository with the dataset identifier PXD031046 and 10.6019/PXD031046.

## Results

### *Tgfbr2* Deletion is Isolated to the DP Mesenchyme

*Tgfbr2* ^*cko*^ mice demonstrated alterations in development of the M1s by P5 (Wang et al., 2013). To investigate the mechanisms responsible for the phenotypes observed in *Tgfbr2* ^*cko*^ teeth, we performed H&E staining on sections from P5, P7, P10 and P14 control and mutant M1s. These time points were chosen because tooth root elongation and dentin mineralization were both actively occurring on these days. Axon-like structures were reduced in mutant teeth relative to controls at all stages (Supplementary Figure S1).

**FIGURE 1:**
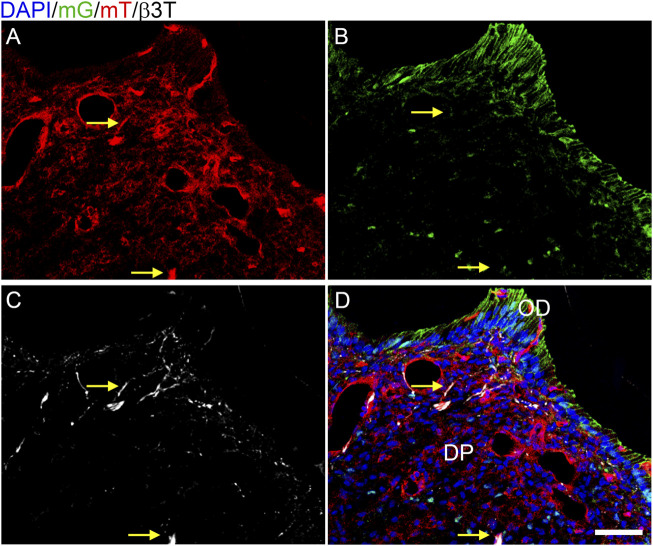
Cre activity is isolated to the mesenchyme in P7 Osterix-Cre;ROSA26mTmG mouse dental pulp. Z-stacks (10 μm thick) were collected and presented in maximum projection mode to examine the Cre expression in DP structures. **(A)** membranous tomato red (mT) indicating an absence of Cre activity; **(B)** membranous green (mG) and nuclear Osterix-GFP indicating Cre activity; **(C)** β3T immunofluorescence labelling axonal afferents in white. The β3T correlated with only mT **(A–D)** and never mG **(B–D)**, demonstrating that Cre activity is isolated to the DP mesenchyme and odontoblasts (OD). Arrows highlight two particular regions of β3T and mT without mG, with a dense β3T+ structure indicated in the bottom center and a single-fiber β3T+ structure in the top center **(C)**. N = 8 Osterix-Cre;ROSA26mTmG mice. The scale bar is 50 μm.

We subsequently assessed whether the hypothesized reduction of innervation in the *Tgfbr2* ^*cko*^ mice was due to unexpected Cre activity in neurons by crossing Osterix-Cre and ROSA26-mTmG reporter mice. The ROSA26-mT/mG reporter line expresses a membrane-bound GFP (mG) tag in cells that express Cre at any point, or a Tomato red fluorescent (mT) tag in cells that never express Cre (Muzumdar et al., 2007). Osterix:GFP:Cre is also expressed in the nucleus (Rodda and McMahon, 2006). We collected P7 Osterix-Cre;ROSA26mTmG DP and performed immunofluorescence staining for β3T on 10 μm cryosections with a Cy5 secondary antibody, followed by confocal microscopy for mG, mT, and Cy5 to detect neuronal afferents. We found scattered nuclear GFP in the DP and membranous GFP (mG) in the odontoblast (OD) layers (Figures 1B,D), with mT isolated to DP cells without nuclear GFP (Figures 1A,D). These results agree with previous reports of Osterix-Cre being isolated to the odontoblast and DPC populations within the developing tooth (Rakian et al., 2013; Wang et al., 2013; Ono et al., 2016). There was no co-labelling of mT and mG, as expected (Muzumdar et al., 2007). The β3T labelling of microtubules in the neuronal afferents did not correlate to any GFP+ structures and localized in some regions with the membranous mT. This confirmed that Osterix-Cre was isolated to the DP mesenchymal lineage and that there was no off-target deletion of *Tgfbr2* in the axonal afferents of *Tgfbr2* ^*cko*^ M1s.

### Afferent Innervation is Reduced in *Tgfbr2* ^*cko*^ Molars

**FIGURE 2:**
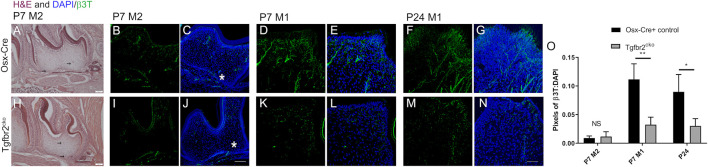
DP innervation is reduced in developing *Tgfbr2* ^*cko*^ molars. Innervation did not appear significantly disrupted during early postnatal tooth development. Representative images of H&E-stained mandibular M2s at P7 **(A–H)** with two black arrows in each image indicating hypothesized neuronal structures. Scale bar = 100 μm. *N* = 3 for control Osterix-Cre and 4 *Tgfbr2* ^*cko*^ samples. **(B–G, I–N)** Z-stacks (10 μm thick) of β3T-positive structures were collected and presented in maximum projection mode to demonstrate total afferent innervation. **(B–C, I–J)** Confocal images of control **(B–C)** and *Tgfbr2* ^*cko*^ **(I–J)** M2 DP demonstrated afferent structures throughout the entire tooth. The asterisk shows similar areas of nerve penetration in the apical region of the tooth. The scale bar is 100 μm. Coronal neurite outgrowth appeared to be more extensive in control mice, but the overall afferent structures were not significantly different between control and *Tgfbr2* ^*cko*^ mice as indicated by unpaired *t*-tests **(O)**. **(D–G, K–N)** Confocal images of control **(D–G)** and *Tgfbr2* ^*cko*^ **(K–N)** M1 DP demonstrated that there were more β3T+ neuronal structures in P7 and P24 control mice than mutant mice. The scale bar is 50 μm. **(O)** The ratios of total pixels for neuronal structures normalized to the pixels for DAPI indicate that there was significantly more innervation per region in control mice than mutant mice at P7 and P24. *, *p* < 0.05, **p < 0.01 by unpaired *t*-tests, *n* = 4 for Osterix-Cre and 4 for *Tgfbr2* ^*cko*^ samples at each time point.

To directly assess innervation, sections from P7 and P24 control and mutant molars were stained with pan-neuronal β3T, and the levels of fluorescence were compared. Nerve fascicles branched throughout both the control and mutant DP, with individual axons entering the predentin and dentin regions of the tooth organ (Figure 2). Maximum projections from confocal microscopy were used to evaluate and quantify fluorescence as a measure of tooth innervation. The total axon coverage was quantified as the pixel density of equivalent regions of the image normalized to DAPI. We found initial nerve penetration in both control and *Tgfbr2* ^*cko*^ second molars (M2s), with no significant difference in total innervation (Figure 2O). To investigate the difference between nerve penetration and neurite outgrowth, we compared the levels of β3T fluorescence in the densely innervated coronal regions of P7 and P24 control and mutant first molars. Nerve fascicles branched throughout both the control and mutant DP, with individual axons entering the predentin and dentin regions of the tooth organ (Figures 2D–G,K–N). We found a significant reduction in nerve fiber coverage in molars from P7 and P24 *Tgfbr2* ^*cko*^ mice compared to controls (Figure 2O), supporting the hypothesis that *Tgfbr2* in the DP mesenchyme mediates tooth innervation. Similar nerve penetration was seen in the M2s, and that observation in tandem with the persistent low amount of innervation in *Tgfbr2* ^*cko*^ mice at P7 and P24, suggest that this phenomenon was not due to a developmental delay, but that the cellular mechanisms guiding neurite outgrowth in developing teeth were disrupted by the deletion of *Tgfbr2*. Since *Tgfbr2* ^*cko*^ mice die around 4 weeks of age (Wang et al., 2013; Peters et al., 2017), we were unable to investigate beyond this time point.

### Vascularization is Not Altered in *Tgfbr2* ^*cko*^ Molars

**FIGURE 3:**
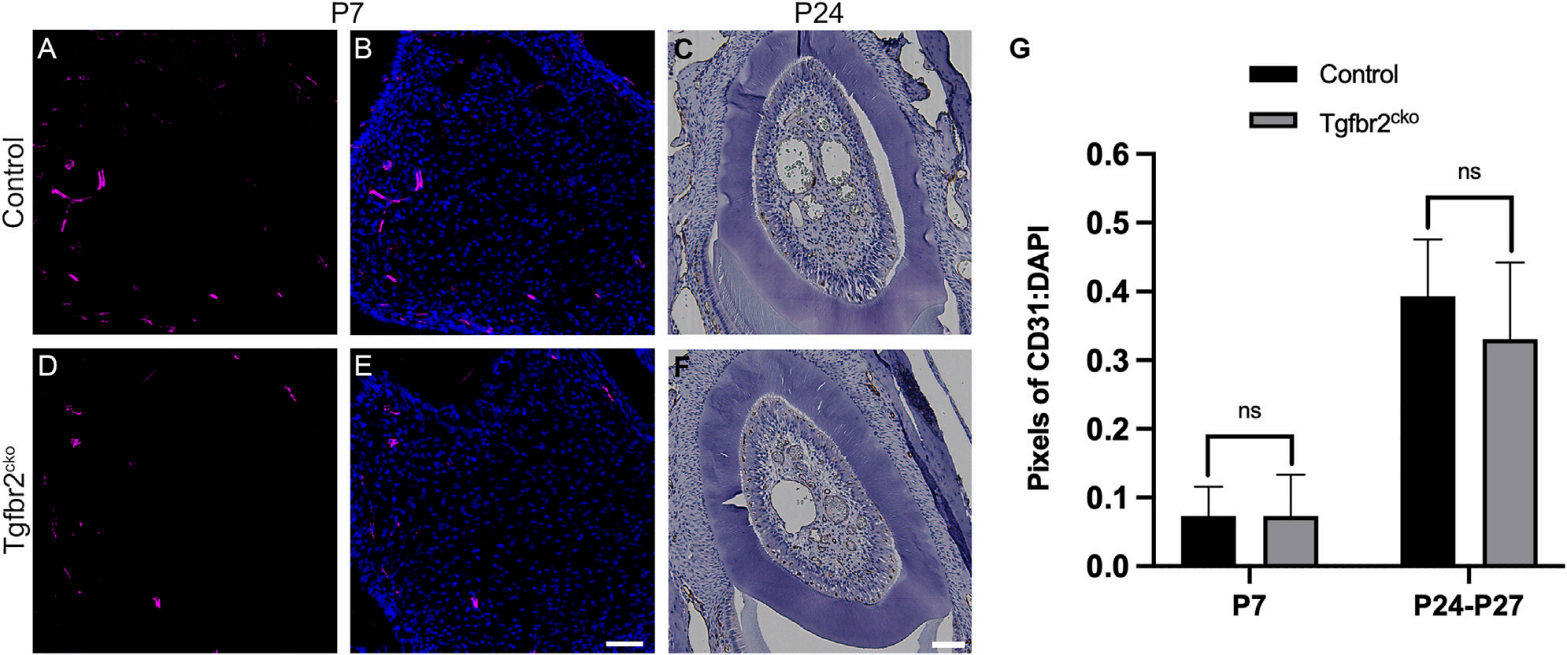
Vascularization is not significantly different between *Tgfbr2* ^*cko*^ and control Osterix-Cre P7 and P24-27 first molars. Representative images of vascular structures labelled by CD31 in P7 and P24 control and *Tgfbr2* ^*cko*^ M1s. **(A,B,D,E)** Representative confocal images of vasculature structures amidst DP cells (nuclei labelled by DAPI) in sagittal sections of P7 Osterix-Cre control **(A,B)** and *Tgfbr2* ^*cko*^ **(D,E)** first molars. **(C–F)** Representative brightfield images of CD31 IHC in horizontal sections of P24 control **(C)** and *Tgfbr2* ^*cko*^ **(F)** first molars. For quantification, confocal images **(A,B,D,E)** were thresholded using FIJI software to remove background fluorescence and the remaining pixels were quantified and normalized to DAPI. For P24-P27 IHC images **(C–F)**, color deconvolution using FIJI preceded thresholding and quantification. For both time points, an unpaired *t*-test showed no significant difference between genotypes **(G)**. For P7, *n* = 3 for each genotype; for P24-P27, *n* = 5 for control and 6 for *Tgfbr2* ^*cko*^. The scale bars are 50 µm.

Blood vessels and nerve fibers often run along parallel tracks with similar branching patterns in developing tissues [reviewed in (Carmeliet, 2003; Serini and Bussolino, 2004; Adams and Eichmann, 2010)]. We found that the β3T+ afferents in some regions resembled vasculature in the Osterix-Cre;ROSA26-mTmG mouse molars, making it difficult to determine whether *Tgfbr2* deletion might also be reducing tooth vascularization. To address this possibility, we assessed vascularization in P7 and P24-27 control and mutant molars using CD31/PECAM immunofluorescence and IHC labelling, respectively. Because the tooth organ is much larger at P24, we sectioned the tissue on the horizontal plane and captured images at a lower objective to obtain a more comprehensive view of the vascularization. Figure 3 shows that there were no significant alterations in vascularization at either time point. These results confirm that tooth vascularization and innervation occur *via* different mechanisms during development (Shadad et al., 2019) and that this distinction remains intact within the *Tgfbr2* ^*cko*^ mice.

### Downregulation of Osteogenic Genes in *Tgfbr2* ^*cko*^ Molars Confirms Hypomineralization Phenotype

**FIGURE 4:**
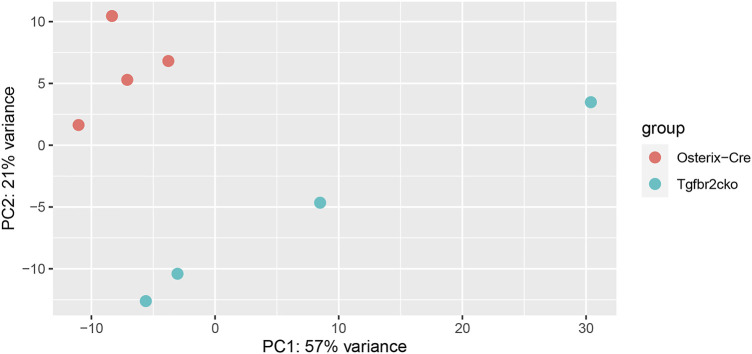
Results of principal components analysis (PCA) of RNA-Seq data from Osterix-Cre and *Tgfbr2^cko^* DP at P7 for all samples used in this study.

To investigate the mechanisms responsible for the phenotypes observed in *Tgfbr2* ^*cko*^ teeth, a global analysis of gene expression was performed using RNA-Seq. We chose to perform this analysis at P7 because this was the earliest time point at which mineralization and innervation differences were apparent. A principal components analysis (PCA) was performed to cluster the samples associated with mouse genotypes to confirm the differences between the Osterix-Cre and *Tgfbr2* ^*cko*^ samples (Figure 4). Select genes from the RNA-Seq analysis were confirmed by qPCR (Supplementary Table S2). The expression of a specific transcript was considered to be altered in *Tgfbr2* ^*cko*^ tissue if the expression differed by at least ±1.5-fold relative to control with *p* < 0.05. These studies showed that several genes associated with osteo/odontogenesis and mineralization were dysregulated by *Tgfrbr2* knockout. Our data confirmed the downregulation of select genes known to be associated with odontogenesis and mineralization: *Bglap*, *Bmp1*, *Col1a1*, *Nes*, and *Wnt10a* (Gorter de Vries et al., 1987; About et al., 2000; Yamashiro et al., 2007; Quispe-Salcedo et al., 2012; Bae et al., 2015; Andersson et al., 2017; Zhang et al., 2017; Foster et al., 2018; Niwa et al., 2018; Rosowski et al., 2019). The expression of DSPP trended toward downregulation as well, with *p* = 0.05. We also confirmed a lack of dysregulation of *Dmp1* and *NFIC* (Supplementary Figure S2)*,* as previously reported (Wang et al., 2013). Clusterin (*Clu*), a gene reportedly regulated by TGFβ signaling during postnatal tooth development (Khan et al., 2013), was upregulated in the mutant mice. There was also upregulation of Fibulin-7 (*Fbln7*), a gene involved in cellular adhesion during predentin and dentin formation (de Vega et al., 2007; Arany et al., 2009), in the *Tgfbr2* ^*cko*^ dental pulp compared to control teeth. Lastly, Tenascin N (*Tnn*) was upregulated, indicating a lack of differentiation of the dental pulp into mineralizing odontoblasts (Thesleff et al., 1987; Sahlberg et al., 2001; Imhof et al., 2021). Together, these data indicate that *Tgfbr2* deletion led to developmental disruption resulting in tooth hypomineralization, confirming the previous reports (Wang et al., 2013; Peters et al., 2017; Corps et al., 2021).

### Neuronal Genetics are Disrupted in *Tgfbr2* ^*cko*^ Molars

**FIGURE 5:**
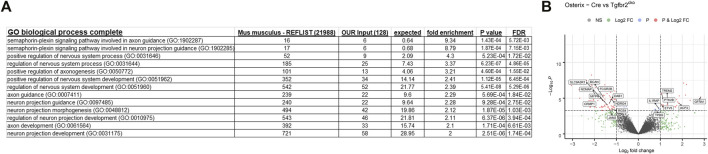
Panther gene ontology analyses identify several transcriptomic categories related to axon guidance that differ between the Osterix-Cre and *Tgfbr2* ^*cko*^ mice. **(A)** Several GO biological complete categories were identified as being over-represented based on the transcriptomes that were dysregulated by ≥1.5-fold with *p* < 0.05 in *Tgfbr2* ^*cko*^ mice, as identified by RNA-Seq. The Panther gene ontology analysis was performed using the Panther GO biological process complete for overrepresented genes with a Fisher’s exact test. **(B)** A Volcano plot of the RNA-Seq data demonstrating significant differences between Osterix-Cre and *Tgfbr2* ^*cko*^ samples and select overrepresented neuronal genes reported by Panther in the categories shown in **(A)**.

A gene ontology analysis was performed using the lists of regulated transcripts (≥1.5-fold difference, *p* < 0.05) generated above. The analysis was performed using the Protein Analysis Through Evolutionary Relationship (Panther) biological processes tool (www.pantherdb.org) (Mi et al., 2010). This tool determines which genes are over-represented in a specific dataset relative to the whole transcriptome. Several neuronal transcriptomes were over-represented. The neuronal bodies that innervate teeth are located in the trigeminal nerve bundle outside of the teeth, so only axonal projections could contribute to the neuronal RNA in the DP. We therefore concentrated on the biological categories related to axon projection and guidance, and the findings are presented in Figure 5A. There were several over-represented neuronal transcripts identified by Panther, with select transcripts shown in the volcano plot of genes differentially expressed between Osterix-Cre and *Tgfbr2* ^*cko*^ samples (Figure 5B). The entire list of over-represented transcripts from these categories is presented in Supplementary Table S3.

We did not observe dysregulation of well-known neurotrophins, brain-derived neurotrophic factor (*Bdnf*), glial-cell derived neurotrophic factor (*Gdnf*), nerve growth factor (*Ngf*) *or neurotrophin 3 (NT-3)* in our RNA-Seq analysis (Supplementary Figure S2)*,* despite several reports that these neurotrophins are released by DP cells and dentin to promote neurite outgrowth (de Almeida et al., 2014; Martens et al., 2014; Austah et al., 2019; Widbiller et al., 2019; Pagella et al., 2020; Sultan et al., 2020)*.* Because this was surprising, we performed subsequent qPCR experiments to verify these findings. These experiments confirmed that there were no significant alterations of *Bdnf*, *Gdnf*, or *Ngf* in the *Tgfbr2* ^*cko*^ samples but confirmed downregulation of *Mypn* with qPCR (Supplementary Table S2). Myopalladin is structurally similar to palladin (Otey et al., 2005), a gene highly associated with axonal growth and maintenance (Boukhelifa et al., 2001; Hwang et al., 2001). Our RNA-Seq analysis also indicated that there was downregulation of several other neuronal transcripts. Synaptotagmin 5 (*Syn5*) and 15 (*Syn15*)*,* which belong to the family of neurotransmitter release genes in axons (Norlin et al., 1999; Dean et al., 2012; Südhof, 2013), were both downregulated in the *Tgfbr2* ^*cko*^ mice. There was also downregulation of *Fxyd2, Fxd6, TMEM178A, TMEM47, TMEM88, TMEM94, TMEM97,* and *TrpM3*, all genes that were previously reported to be specifically expressed in the axonal afferents of mouse molars (Emrick et al., 2020). We confirmed our RNA-Seq could detect alterations in axonal-specific RNA by performing immunofluorescence and confocal imaging for MYPN. We found very specific labelling of MYPN in the axonal structures (Supplementary Figure S3). Thus, there was a reduction in the expression of numerous genes associated with axonal transcriptomes in mutant mouse molars, confirming the phenotypic reduced afferent innervation in the *Tgfbr2* ^*cko*^ mouse molars.

### Neurite Outgrowth in DP-TGN Co-Cultures Requires *Tgfbr2*

**FIGURE 6:**
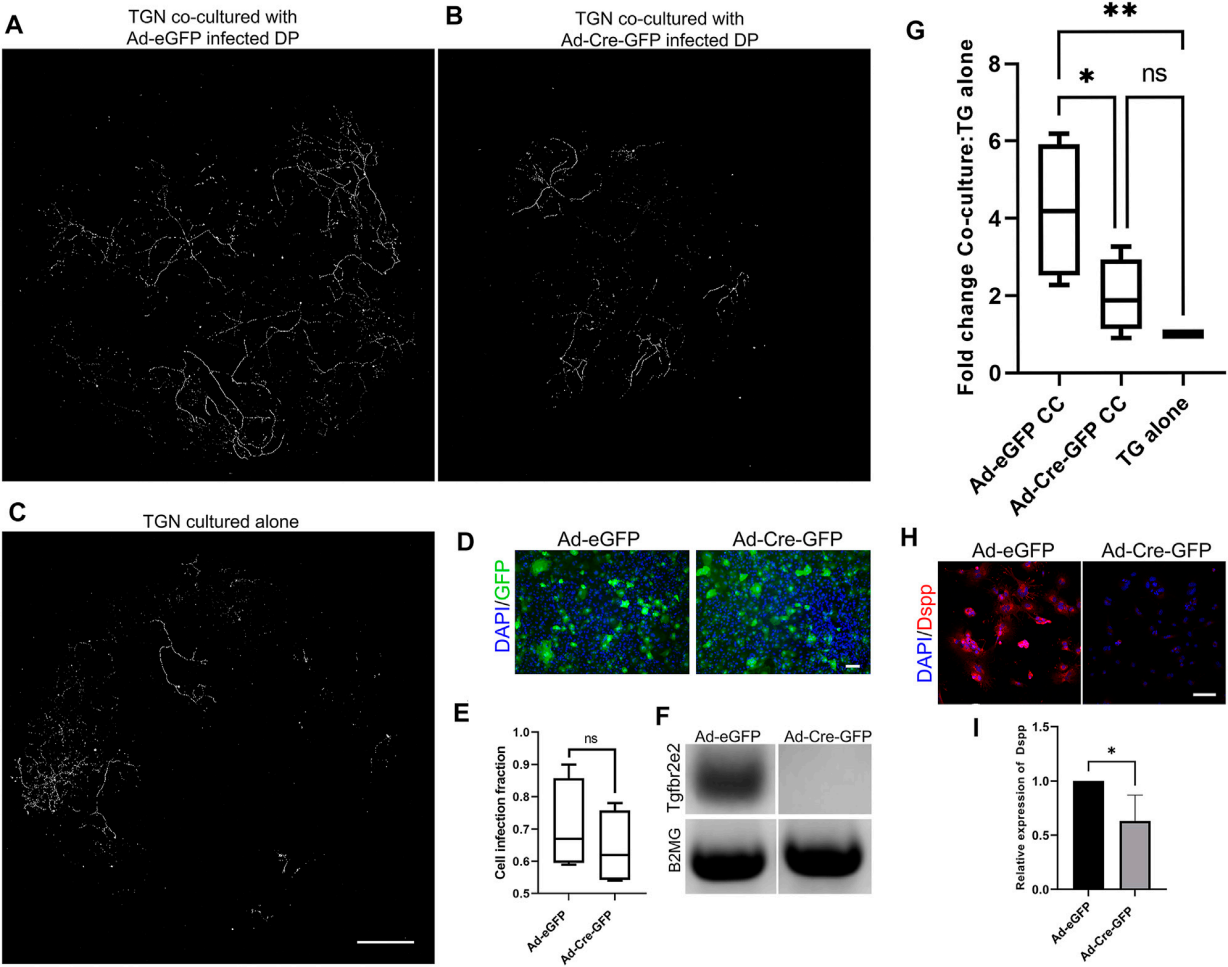
*Tgfbr2* signaling in primary DP mediates trigeminal neurite outgrowth in co-culture. **(A–C)** Thy1-YFP trigeminal neurons were cultured in Transwell filters with 3 μm pores atop primary *Tgfbr2* ^f/f^ DPCs. Immunofluorescence was performed for the YFP protein using an anti-GFP antibody for highly specific staining of neuronal structures over the entire filter. Maximum projections of 100 μm z-stack confocal microscopy images at 10× were collected and stitched with stitching software. TG neurons demonstrated significantly more outgrowth when co-cultured with DPCs **(A)** than when cultured alone **(C)**. Neurite outgrowth was not induced when neurons were co-cultured with DP infected with Ad-Cre-GFP to knock down *Tgfbr2* **(B)**. The scale bar is 1,000 μm for **(A–C)**. **(D)** Adenovirus infection was confirmed with GFP expression and normalized to DAPI to confirm equivalent numbers of infected cells between control Ad-GFP and Ad-Cre-GFP DPCs **(D,E)** The scale bar is 100 μm for **(D)**. Semi-quantitative PCR confirmed *Tgfbr2* KD **(F)**. Images of axonal outgrowth on the filters were auto-thresholded with FIJI software to remove background fluorescence and the remaining pixels were quantified and normalized to the TGN cultured alone from each experimental set **(G)**. *p <0.05, **p < 0.01 by 2-way ANOVA tests with a Tukey’s multiple comparison post-hoc set. **(H)** Representative images of DPCs from co-culture demonstrating reduced expression of an odontoblast marker, DSPP. **(I)** Quantification of DSPP normalized to DAPI demonstrates a significant reduction in cells with *Tgfbr2* deletion. *p < 0.05 by two-tailed unpaired *t*-test. All analyses are based on four separate experiments.

We utilized co-cultures to directly assess the role of *Tgfbr2* in neurite outgrowth in response to DP secretions (Figure 6). Transwell filters were seeded with TG cells, and DPCs were grown in the bottom wells. TGN from Thy1-YFP mice were used so that neurite outgrowth through the filter could be easily imaged. It was previously reported that Thy1-YFP levels become detectable around P6-P10 in neurons and increases exponentially throughout the nervous system during postnatal and adult life (Caroni, 1997; Alić et al., 2016). We observed the brightest staining of Thy1-YFP axons (using the cross-reactive GFP antibody) at 6 weeks of age, so TGN from 6-weeks old mice were used in our co-culture experiments. We performed confocal microscopy and image stitching of the axonal extensions throughout the entire filter and quantified auto-thresholded images. When the TGN were grown on filters in the absence of DPCs, very little neurite outgrowth occurred in comparison to when the TGN were grown above control DPCs (Figures 6A,C,G), confirming that PCs secrete paracrine factors that promote neurite growth (de Almeida et al., 2014; Pagella et al., 2020; Sultan et al., 2020). To examine whether *Tgfbr2* from the DPCs regulates neurite outgrowth, *Tgfbr2* was deleted using Ad-Cre-GFP. Cells infected with Ad-GFP were used as controls. Equivalent numbers of infected cells were used (as identified by GFP fluorescence; Figures 6D,E), and deletion of *Tgfbr2* was confirmed by semi-qPCR (Figure 6F). Deletion of *Tgfbr2* in the DPCs significantly decreased the neurite extension to a level similar to cultures without any DPCs (Figures 6A–C,G). We used DSPP as a marker to confirm that there was reduced odontoblast differentiation and mineralization (Chen et al., 2009; Dissanayaka et al., 2012; Hou et al., 2012) in cultures with Tgbr2 deletion in the DPCs due to Ad-Cre infection (Figures 6H,I). We collected media from the co-culture wells, TG cells cultured alone, and DP cells cultured alone (DPA) with and without *Tgfbr2* deletion for proteomics analysis. Similar to our RNA-Seq data, we did not find significant alterations in NGF, BDNF, GDNF, or NT-3 (data not shown), indicating *Tgfbr2* did not regulate secretion of these neurotrophins. The results of the co-culture experiments suggest that *Tgfbr2* in the DP mesenchyme is required to promote both odontoblastic differentiation and subsequent neurite outgrowth.

### *In Vivo* and *In Vitro* Models Suggest Odontoblast Differentiation Coordinates Axonal Guidance Cues

**FIGURE 7:**
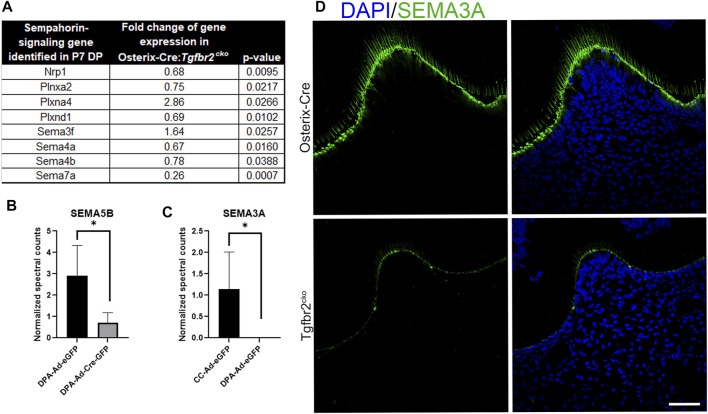
*Tgfbr2* deletion in DP cells alters semaphorin signaling. **(A)** RNA Seq analysis of P7 M1 tissue indicates altered semaphorin signaling components. *N* = 5 for Osterix-Cre and *Tgfbr2* ^*cko*^ samples. **(B,C)** Proteomic analysis of co-culture (CC) and dental pulp alone (DPA) media. **(B)** Proteomics indicates decreased Semaphorin 5B present in the media with *Tgfbr2* deletion in the DPCs. **(C)** Proteomics indicates Semaphorin 3A is only present in CC media and not when dental pulp cells were cultured alone. *N* = 4 media collected and analyzed from experiments including: co-culture samples with and without *Tgfbr2* deletion, DPA samples with and without *Tgfbr2* deletion, and TG cells cultured alone. *p < 0.05 by unpaired *t*-test. **(D)** Immunofluorescence of SEMA3A (green) demonstrates reduced chemoattraction signals in the *Tgfbr2* ^*cko*^ odontoblasts compared to controls. *N* = 4 for Osterix-Cre and 4 *Tgfbr2* ^*cko*^ samples. The scale bar is 50 μm.

We next searched the RNA-Seq data from the P7 M1s and proteomics data obtained for co-culture media to identify neurotrophic factors that were downregulated by *Tgfbr2* deletion. We found that both the RNA-Seq and proteomics analyses identified several semaphorins, which are well-known axonal guidance cues (Kitsukawa et al., 1997; Lillesaar and Fried, 2004; Ding et al., 2007; Fried et al., 2007; Kolk et al., 2009; Sijaona et al., 2012; Moe et al., 2012; Sabag et al., 2014; Shrestha et al., 2014; Yoshida et al., 2016; Song et al., 2017; Yu et al., 2018; Bott et al., 2019; Coutiño and Mayor, 2021), were dysregulated with *Tgfbr2* deletion (Figure 7). Our RNA-Seq identified *Sema3f*, *4a*, *4b*, and *7a* were altered in the *Tgfbr2* ^*cko*^ molars. The RNA-Seq also identified dysregulation of semaphorin receptors, Plexin A2 (*PlxnA2*), Plexin A4 (*PlxnA4*), Plexin D1 (*PlxnD1*) and Neuropilin 1 (*Nrp1*) (Sijaona et al., 2012; Sabag et al., 2014; Yoshida et al., 2016; Song et al., 2017; Limoni, 2021) when *Tgfbr2* was deleted (Figure 7A). *Sema3a* was expressed at levels too low for RNA-Seq statistics. We observed reduced Semaphorin 5B (SEMA5B) secretion by the DPCs into the media with *Tgfbr2* deletion when they were cultured alone, suggesting that it is directly regulated by *Tgfbr2* signals in the DPCs (Figure 7B). Interestingly, we did not find any Semaphorin 3a (SEMA3A) in the media of DPCs cultured alone whether or not *Tgfbr2* was deleted (Figure 7C). These data suggest that the complex interplay between neuronal and mesenchymal cells could be regulated through coordinated semaphorin signaling.

We next questioned whether the reduced differentiation of the developing teeth and DPCs with *Tgfbr2* knockout could be disrupting the necessary signals to support neurite outgrowth. Semaphorin signaling is known to regulate non-neuronal functions, including odonto-/osteogenic differentiation (Sijaona et al., 2012; Song et al., 2017; Yu et al., 2018), so we performed immunoflouresence for SEMA3A on P7 Osterix-Cre and *Tgfbr2* ^*cko*^ M1s to determine which cell population(s) demonstrated altered semaphorin signaling. We found reduced SEMA3A protein levels in the odontoblast layer of the *Tgfbr2* ^*cko*^ mice compared to the controls. Neither mouse genotype demonstrated detectable levels SEMA3A in the pulpal cells (Figure 7D), indicating that axonal guidance in the coronal regions of the teeth may be orchestrated by the odontoblast population.

In summary, our data demonstrate that deletion of *Tgfbr2* in the DP of mice during TGN sprouting in the tooth changes inherent axonal guidance cues, resulting in reduced innervation in postnatal teeth. Co-culture experiments supported that *Tgfbr2* in the DP is required for odontoblast differentiation and DP-mediated neurite outgrowth. We propose that *Tgfbr2* regulates the signals necessary to promote the mineralization and innervation of the tooth organ into a hard tissue capable of the daily activities of the oral cavity.

## Discussion

Tooth innervation serves unique roles that allow teeth to sense pressure, temperature, and infectious agents. Without these sensations, decay and normal oral activities would cause irreparable damage to the tooth and surrounding tissues. Teeth are primarily innervated by nociceptors extending from the trigeminal ganglia (Fried et al., 2007) that respond to signals from the dental mesenchyme during development (Lillesaar et al., 1999; Fried et al., 2005, 2007; Kettunen, 2005; Kettunen et al., 2007; Moe et al., 2012; Yu et al., 2012; de Almeida et al., 2014; Pagella et al., 2020; Sultan et al., 2020). Interestingly, the DP mesenchyme initially secretes repellant signals during embryogenesis to prevent axon entry into the developing tooth organ, which later shifts to the secretion of attraction factors as the tooth nears eruption (Lillesaar and Fried, 2004; Fried et al., 2007). While most organs are fully functional and innervated by the time of birth, tooth development extends into adult life, with tooth innervation and mineralization occurring in concert during different postnatal stages (Kollar and Lumsden, 1979; Moe et al., 2012). These previous reports clearly demonstrate that neuronal-mesenchymal crosstalk is required for tooth organogenesis and maintenance, but the details of these mechanisms remained largely unclear. We herein present the first *in vivo* and *in vitro* data showing neurite responses to the deletion of *Tgfbr2* in the DP mesenchyme during postnatal development. Our results delineate a novel role for *Tgfbr2* in the DP mesenchyme, where it regulates the development of sensory activity in teeth.

Of interest, neither our RNA-Seq nor proteomics data uncovered any significant alterations in NGF, BDNF, GDNF, or NT-3, which were previously reported to correlate with neurite outgrowth (Lillesaar et al., 2001; Lillesaar and Fried, 2004; Price et al., 2005; Kitisin et al., 2007; Austah et al., 2018, 2019; Widbiller et al., 2019; Pagella et al., 2020). The differences in the model systems used may underlie many of the observed differences. For example, some groups have used cultures of specific cell populations [e.g., stem cell apical papilla (SCAP) cells (de Almeida et al., 2014) or DPCs from rat incisors (Sultan et al., 2020)], while we included *in vivo* and *in vitro* studies of the entire pulp tissue from the molar tooth organ, including the odontoblasts. The different findings may reflect the differences in cell-cell interactions, the specific media and additives used for culture, or the differences between the *in vitro* and *in vivo* cellular milieu. It is also possible that the impact on the semaphorin pathway may have been overlooked in the previous studies due to their focus on the changes in the above markers. Our assays investigated global expression at both the RNA and protein levels, permitting the identification of novel mediators of neurite outgrowth.

We reported that several genes associated with odontoblast differentiation and mineralization were downregulated in the *Tgfbr2* ^*cko*^ DP, verifying previous reports of hypomineralized dentition when *Tgfbr2* was knocked out (Wang et al., 2013; Corps et al., 2021). We also found upregulation of some genes related to mineralization, such as *Fbln7, Clu,* and *Tnn*. *Fbln7* is expressed by preodontoblasts and odontoblasts in fibronectin-rich regions and is involved in dentin formation (de Vega et al., 2007; Arany et al., 2009). The increased expression of *Fbln7* in the *Tgfbr2* ^*cko*^ dental pulp may be present to compensate for the decreased fibronectin-related genes, fibronectin domain containing protein 3a (*Fndc3a*) and fibronectin domain containing protein 3b (Fndc3b) (data not shown), in the *Tgfbr2* ^*cko*^ mice. *Fndc3a* and *Fndc3b* are both also called Factor for adipocyte differentiation 104 (*Fad104*), which is reportedly regulated by TGFβ signaling (Goto et al., 2017) and contributes to bone formation (Kishimoto et al., 2013; Cao et al., 2016). Additional studies are needed to elucidate how *Fbln7* and *Fad104* contribute to the cell-extracellular matrix interactions during tooth development. We also found upregulation of *Clu,* a gene highly upregulated during secretory odontogenesis in early postnatal tooth development (Khan et al., 2013). However, reports that *Clu* is also known to promote axonal outgrowth led us to postulate that the DP could be secreting excess Clusterin in the *Tgfbr2* ^*cko*^ mice in an attempt to overcome the lack of innervation (Kang et al., 2005; Wright et al., 2014). We hypothesized that some of the mineralization-related genes with altered expression levels might instead be regulating axonal outgrowth in the tooth. For instance, Wnt proteins are also known to play important roles in the nervous system. In support of this theory, we found downregulation of *Wnt10a,* which is thought to promote axon growth by activating small G-proteins (Matsukawa et al., 2018). We noted that there was upregulation of *Tnn,* which is often localized with Tenascin-C ( *Tnc*) (Meloty-Kapella et al., 2006). This is particularly interesting because tenascins are developmentally regulated by TGFβ signaling in the tooth (Thesleff et al., 1987; Sahlberg et al., 2001). A recent report indicating that TNN deficiency in dental tissues resulted in increased axonal branching (Imhof et al., 2021) further suggested that tenascins promote neurite outgrowth (Wehrle and Chiquet, 1990; Chiquet and Wehrle-Haller, 1994; Meiners et al., 1999; Rigato et al., 2002; Michele and Faissner, 2009). In addition to our RNA-Seq data, we found that high levels of Tenascin and Tenascin-C were secreted by DPCs in our proteomics study, indicating that tenascins may play a role as a neurotrophic paracrine signals. Together, the phenotypic, genetic and proteomic data suggest that the differentiation of the DP mesenchyme is developmentally regulated, as are axonal guidance cues, and these developmental processes may utilize the same signals.

The semaphorin proteins are well known axonal guidance cues reported to be active in a variety of tissues (Kitsukawa et al., 1997; Pasterkamp et al., 2003; Ding et al., 2007; Kang et al., 2007; Kolk et al., 2009; Kopp et al., 2010; Coutiño and Mayor, 2021; Limoni, 2021) including dental pulp tissues (Lillesaar and Fried, 2004; Maurin et al., 2005; Moe et al., 2012; Sijaona et al., 2012; Shrestha et al., 2014; Kuchler-Bopp et al., 2018). One research group investigated the expression profiles of all the semaphorins in M1s from E13 through P9. They found that *Semaphorin 3a*, *3f*, and *5b* dramatically decreased through the postnatal stages, *Semaphorin 4a* and *4b* remained at high levels in postnatal stages, and *Semaphorin 7a* dramatically increased from P5 to P9 (Lillesaar and Fried, 2004). This highlights the fact that semaphorins do not act in a linear fashion or in isolation, rather that the cooperation of signals regulates the axonal branching that occurs throughout coronal regions of the developing tooth. We found that several semaphorins and semaphorin receptors were altered in both our RNA-Seq and proteomics data. In particular, we saw reduced *Sema4a, Sema4b,* and *Sema7a* in the M1s and increased *Sema3f*. *Sema3a* was expressed at levels below the statistical cutoffs for RNA-Seq, but our immunofluorescence studies indicated stable protein levels in odontoblast cells that were clearly reduced in the *Tgfbr2* ^*cko*^ M1s. Our proteomic analyses indicated that DPCs do not secrete SEMA3A in the absence of TG cells, which highlights the importance of mesenchymal-neuronal populations during development. However, SEMA5B secretion was directly downregulated in isolated DPCs with *Tgfbr2* deletion, suggesting that class 5 semaphorins may be more directly regulated by TGFβ signaling. Of particular note is that the *Tgfbr2* ^*cko*^ DP showed downregulation of *Sema7a*, which is reportedly regulated by TGFβ signals (Kang et al., 2007) to promote axon outgrowth (Pasterkamp et al., 2003). A previous study reported that the SEMA7A localization in the odontoblast layer correlated with axonal branching into the dentin-pulp complex. Co-cultures with transfected cells overexpressing *Sema7a* demonstrated increased neurite outgrowth toward the ganglia explant (Maurin et al., 2005). Our present study suggests the decreased *Sema7a* (and potentially other semaphorins) in our mutant mouse DP mesenchyme may have directly led to decreased axonal outgrowth in the dentin-pulp regions. We also saw an increase of *Sema3f* in the *Tgfbr2* ^*cko*^ M1s, which has been reported to act as a chemorepellent along with its receptor, Plexin A4 (*Plxna4*) (Sijaona et al., 2012)*.* Since we also found increased expression of *Plxna4* in our mutant M1s, we propose that this *Sema3f-Plxn4a* increase could be directly repelling axonal afferents in the *Tgfbr2* ^*cko*^ mice. Previous reports indicated that there is developmental regulation of class 3 semaphorin receptor, Neuropilin (*Nrp1*) (He and Tessier-Lavigne, 1997; Kitsukawa et al., 1997; Sijaona et al., 2012). We observed downregulation of *Nrp1*, which could reflect reduced SEMA3A signaling, particularly because the expression of the other *Sema3a* receptor, Plexin-A2 (*Plxna2*) (Sabag et al., 2014) and SEMA3A levels were also reduced in our mutant mice. Our current findings and previous publications suggest that semaphorin signaling plays an important role in neurite outgrowth throughout the coronal regions of the teeth.

Lastly, previous research indicates that semaphorin signaling is tied into odontoblastic differentiation, with SEMA7A and SEMA3A levels elevated in odontoblast regions (Maurin et al., 2005; Quispe-Salcedo et al., 2012). Exogenous SEMA3A has been shown to enhance osteogenesis (Yu et al., 2018) and odontogenesis (Yoshida et al., 2016) *in vitro*. Similarly, the Class III Semaphorin receptor, NRP1, stimulates odontogenesis *in vitro* (Song et al., 2017)*.* We found that SEMA3A is reduced specifically in the odontoblast layer of our *Tgfbr2* ^*cko*^ *mice* (Figure 8), suggesting this may relate to the downregulated odontoblast differentiation with *Tgfbr2* deletion in the dental mesenchyme. We hypothesize that semaphorin signaling may serve as a feedback mechanism for interactions between the DP mesenchyme and neuronal afferents. Future studies could include several co-culture semaphorin-rescue experiments and investigations into the tooth and bone mineralization in the SEMA7A null mouse. Lastly, in fully developed teeth, axons sprout after dentin injury and then signal to the DP to repair the damaged dentin (Taylor et al., 1988). This suggests that there is a neuronal-mesenchymal feedback loop guiding tooth maintenance that exists into adulthood. Future studies could be performed utilizing the inducible (tet-off) Osterix-GFP:Cre system (Wang et al., 2013) to study whether TGFβ-guided signals, including the semaphorins, are necessary and sufficient to maintain tooth vitality.

In summary, we found that *Tgfbr2* deletion in the DP mesenchyme reduced the ability of DPCs to differentiate and provide the axonal guidance cues necessary to support neurite outgrowth. We propose that investigations of the neuronal structures in other mouse models with hypomineralized dentition would provide information key to understanding peripheral nerve branching and the developmental process of tooth innervation.

## Data Availability

The RNA-Seq data presented in the study are deposited in the NCBI repository, accession number GSE190582. The proteomics data presented in the study are deposited here: http://proteomecentral.proteomexchange.org/. The accession number is PXD031046.

## Supplementary Material

Supplementary Figure S1 Nerve structures in postnatal teeth appear to be reduced in *Tgfbr2* ^*cko*^ mice. Representative images of H&E-stained mandibular M1s from postnatal days 5, 7, 10, and 14. The black arrows in each image indicate hypothesized neuronal structures. Several other structures are present but are not indicated to prevent image crowding. N = 2 for P5 and N = 3 for P7, 10 and P14 for each genotype. The scale bar is 100 μm.

Supplementary Figure S2 A volcano plot of the RNA-Seq data demonstrating that there were no significant differences in select genes between Osterix-Cre and *Tgfbr2* ^*cko*^ samples. The volcano plot visually demonstrates that the *Ngf, Gdnf, Nt-3, Nfic,* and *Dmp1* transcripts were not altered, as reported in the manuscript. *Bdnf* was not reported in the RNA-Seq analysis. RNA-Seq was performed on 4 Osterix-Cre and 4 *Tgfbr2* ^*cko*^ samples.

Supplementary Figure S3 Representative image of Mypn in Osterix-Cre and *Tgfbr2* ^*cko*^ P7 M1. Representative immunofluorescence images of myopalladin in P7 M1 Osterix-Cre and *Tgfbr2* ^*cko*^ mice confirming axonal-specific expression. N = 4 Osterix-Cre and *Tgfbr2* ^*cko*^ samples.
