# Supplementary material for: Tgfbr2 in Dental Pulp Cells Guides Neurite Outgrowth in Developing Teeth
Source: Front Cell Dev Biol. 2022 Feb 21;10:834815. doi: 10.3389/fcell.2022.834815 (PMC8901236; doi:10.3389/fcell.2022.834815)
Supplement: Supplementary file 2 [file Table1.DOCX]

**Supplemental Table 1:** The primers used for the quantitative real-time PCR analysis of DP samples. qPCR was performed on 10 Osterix-Cre and 10 *Tgfbr2^cko^* samples. **Housekeeping gene used for analysis. *Alternative housekeeping genes used for quality assurance for data analyses.

| **Gene Symbol** | **Assay ID** | **assay description** |
| --- | --- | --- |
| **B2MG**** | Mm00437762_m1 | <https://www.thermofisher.com/taqman-gene-expression/product/Mm00437762_m1> |
| **RPL27*** | Mm01245874_g1 | <https://www.thermofisher.com/taqman-gene-expression/product/Mm01245874_g1#genomic-map-section> |
| **Beta-actin*** | Mm02619580_g1 | <https://www.thermofisher.com/taqman-gene-expression/product/Mm02619580_g1#genomic-map-section> |
| **Clusterin** | Mm01197002_m1 | <https://www.thermofisher.com/taqman-gene-expression/product/Mm01197002_m1> |
| **Fbln7** | Mm01336227_m1 | <https://www.thermofisher.com/taqman-gene-expression/product/Mm01336227_m1> |
| **Mypn** | Mm00805430_m1 | <https://www.thermofisher.com/taqman-gene-expression/product/Mm00805430_m1> |
| **NGF** | Mm00443039_m1 | <https://www.thermofisher.com/taqman-gene-expression/product/Mm00443039_m1> |
| **BDNF** | Mm04230607_s1 | <https://www.thermofisher.com/taqman-gene-expression/product/Mm04230607_s1> |
| **GDNF** | Mm00599849_m1 | <https://www.thermofisher.com/taqman-gene-expression/product/Mm00599849_m1> |
