# Supplementary figures and images for: Tgfbr2 in Dental Pulp Cells Guides Neurite Outgrowth in Developing Teeth
Source: Front Cell Dev Biol. 2022 Feb 21;10:834815. doi: 10.3389/fcell.2022.834815 (PMC8901236; doi:10.3389/fcell.2022.834815)

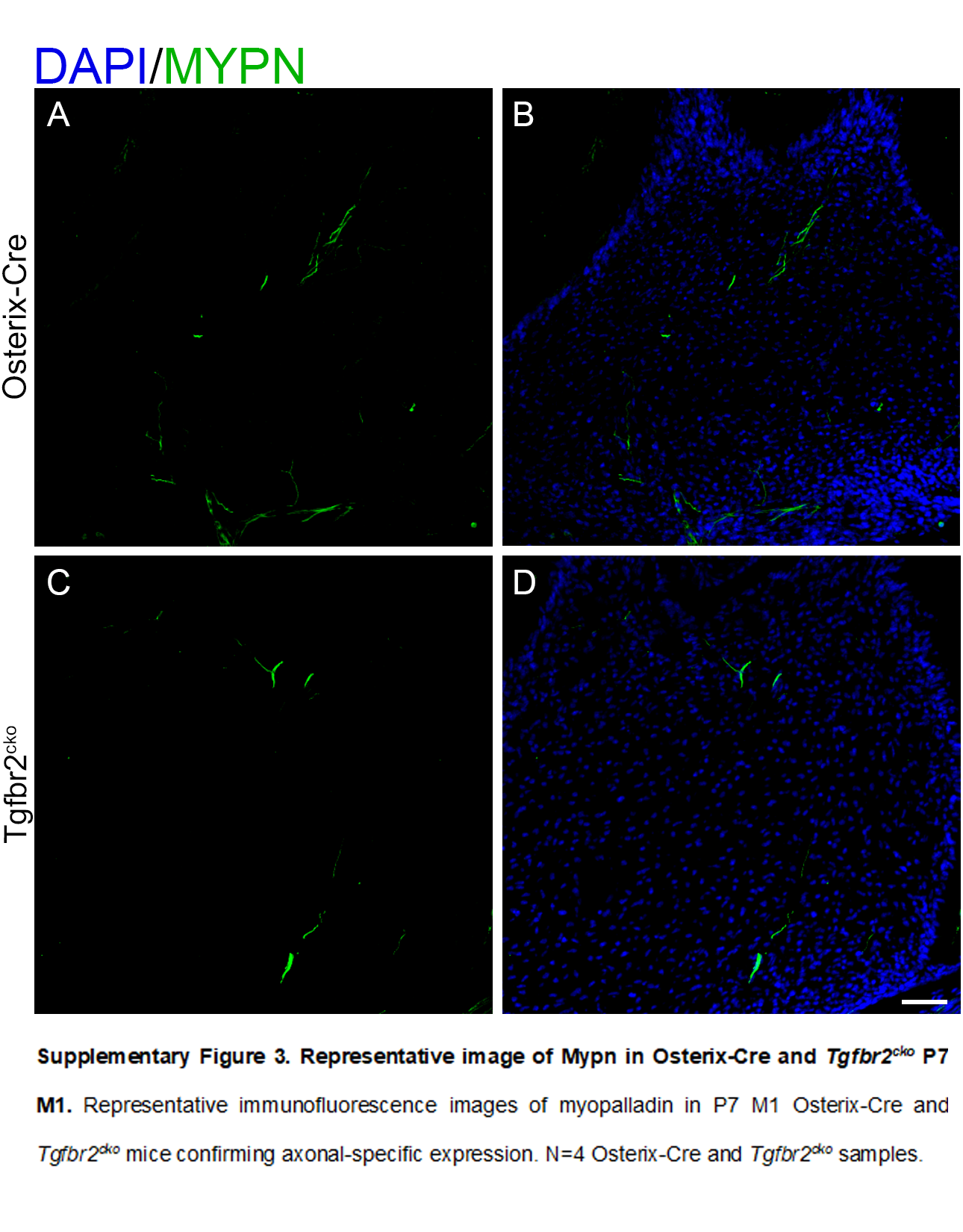

Supplement: Supplementary file 3 [file Image3.tif]

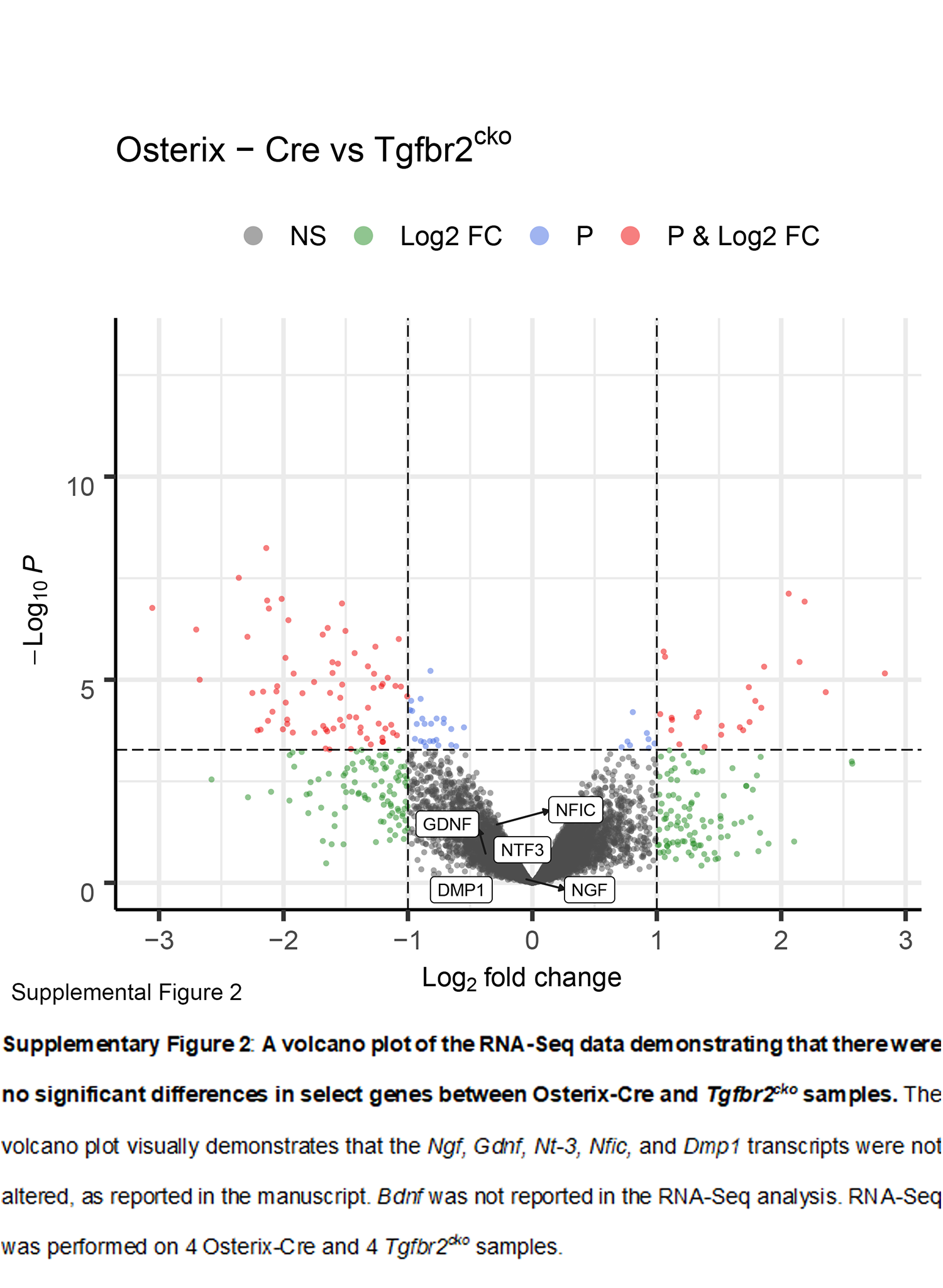

Supplement: Supplementary file 4 [file Image2.tif]

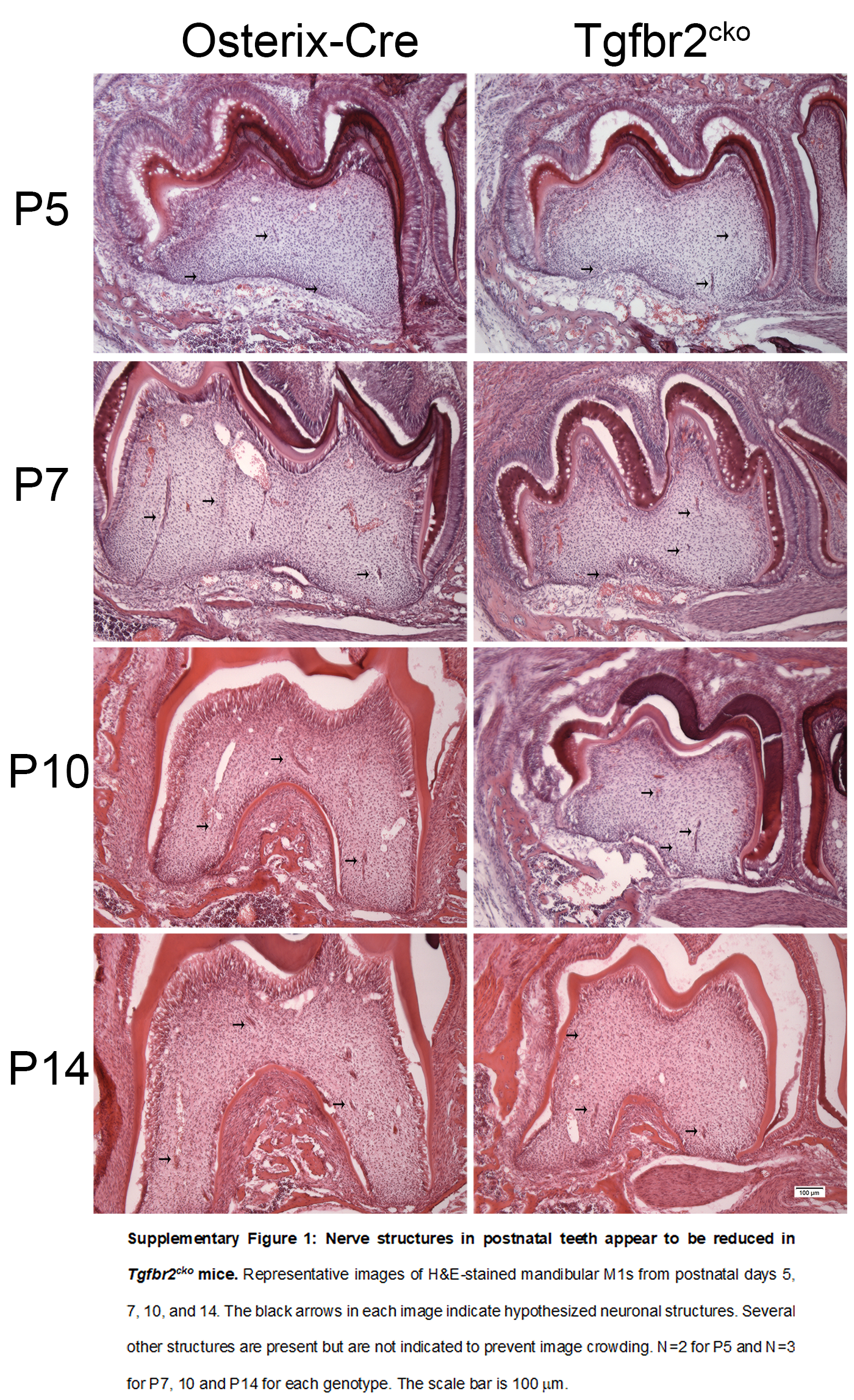

Supplement: Supplementary file 5 [file Image1.tif]
