# Supplementary material for: Tgfbr2 in Dental Pulp Cells Guides Neurite Outgrowth in Developing Teeth
Source: Front Cell Dev Biol. 2022 Feb 21;10:834815. doi: 10.3389/fcell.2022.834815 (PMC8901236; doi:10.3389/fcell.2022.834815)
Supplement: Supplementary file 6 [file Table2.DOCX]

**Supplemental Table 2. Select genes from RNA-Seq verified by qPCR.** Transcriptome alterations identified by the RNA-Seq analysis were verified by qPCR using 10 Osterix-Cre and 10 *Tgfbr2^cko^* samples.

|  | Fbln7 | Clu | Mypn |  | NGF | BDNF | GDNF |
| --- | --- | --- | --- | --- | --- | --- | --- |
| Osterix-Cre:*Tgfbr2^cko^* fold change | 2.47 | 3.36 | 0.39 |  | 0.94 | 4.99 | 0.93 |
| p-value | 0.0035 | 0.014 | 0.000007 |  | 0.69 | 0.27 | 0.74 |
