# Supplementary material for: Tgfbr2 in Dental Pulp Cells Guides Neurite Outgrowth in Developing Teeth
Source: Front Cell Dev Biol. 2022 Feb 21;10:834815. doi: 10.3389/fcell.2022.834815 (PMC8901236; doi:10.3389/fcell.2022.834815)
Supplement: Supplementary file 7 [file Table4.DOCX]

**Supplemental Table 4. Abbreviations used throughout the manuscript.**

**Abbreviations**

Ad-Cre Adenovirus-Cre-GFP

Ad-GFP Adenovirus-eGFP

β3T Class III beta tubulin

BDNF Brain-derived neurotrophic factor

BSA Bovine serum albumin

DP Dental pulp

DPA Dental pulp cells culture alone

DPCs Dental pulp cells

DSPP Dentin sialophosphoprotein

Fad104 Factor for adipocyte differentiation 104

Fndc3a Fibronectin domain containing protein 3a, also Fad104

Fndc3b Fibronectin domain containing protein 3b, also Fad104

GDNF Glial cell derived neurotrophic factor

GFP Green fluorescent protein

H&E Hematoxylin and eosin

IHC Immunohistochemistry

M1 First (mandibular) molar

M2 Second (mandibular) molar

mTmG Membrane tomato membrane GFP

NGF Nerve growth factor

Nrp1 Neuropilin 1

NT-3 Neurotrophin 3

OD Odontoblasts

Osterix-Cre Osterix-Cre-GFP mouse model

P Postnatal day

PCA Principal components analysis

Plxna4 Plexin A4

qPCR quantitative real-time polymerase chain reaction

RNA Ribonucleic acid

RNA-Seq mRNA sequence analysis

SCAP Stem cell apical papilla

TG(N) Trigeminal (neuron)

TGFβ Transforming growth factor beta

Tgfbr2 TGFβ receptor 2

Tgfbr2^f/f^ Tgfbr2 flanked by loxP sites to facilitate gene deletion by Cre recombinase

Tgfbr2^cko^  Tgfbr2 conditional knockout

Tnc Tenascin C

Tnn Tenascin N (also Tenascin W)

WT Wild-type (mouse)
